# Device Applications Enabled by Bandgap Engineering Through Quantum Dot Tuning: A Review

**DOI:** 10.3390/ma17215335

**Published:** 2024-10-31

**Authors:** Ho Kyung Lee, Taehyun Park, Hocheon Yoo

**Affiliations:** 1 Smart Materials Research Center for IoT, Gachon University, 1342 Seongnam-daero, Seongnam 13120, Republic of Korea; hogi0722@gachon.ac.kr; 2 Department of Chemical and Biological Engineering, Gachon University, 1342 Seongnam-daero, Seongnam 13120, Republic of Korea; 3 Department of Semiconductor Engineering, Gachon University, 1342 Seongnam-daero, Seongnam 13120, Republic of Korea; thpark@gachon.ac.kr; 4 Department of Electronic Engineering, Gachon University, 1342 Seongnam-daero, Seongnam 13120, Republic of Korea

**Keywords:** quantum dots, optoelectronics, bandgap tunability

## Abstract

Quantum dots (QDs) are becoming essential materials for future scientific and real-world applications, owing to their interesting and distinct optical and electrical properties compared to their bulk-state counterparts. The ability to tune the bandgap of QDs based on size and composition—a key characteristic—opens up new possibilities for enhancing the performance of various optoelectronic devices. These advances could extend to cutting-edge applications such as ultrawide-band or dual-band photodetectors (PDs), optoelectronic logic gates, neuromorphic devices, and security functions. This paper revisits the recent progress in QD-embedded optoelectronic applications, focusing on bandgap tunability. The current limitations and challenges in advancing and realizing QD-based optoelectronic devices are also discussed.

## 1. Introduction

Quantum dot (QD)-based materials allow facile adjustments of the energy bandgap because of the size-dependent bandgap tunability, making them particularly useful in optoelectronic applications [1]. The most common applications include those involving the interaction between light energy and electrical energy, such as solar cells [2,3,4], photodetectors [5,6,7], light-emitting devices [7,8,9], and lasers [10], or devices where conversion between optical and electrical signals is essential. The ability to tune the bandgap, which determines the wavelength of light that can be absorbed, is a significant advantage of QD materials. QDs have intermediate properties between film or bulk semiconductors and discrete atoms, meaning their optoelectronic properties vary depending on the size and shape. For instance, when the size of QDs decrease, the bandgap energy of them will be enlarged, resulting in shorter wavelength photon irradiation. On the other hand, the bandgap of the QDs will be narrowed if the size of them increases, leading to much longer wavelength emission. Recently, based on the size-dependent bandgap tunability of QDs, considerable efforts have been made to develop new application devices beyond conventional uses in the aforementioned solar cells, photodetectors, LEDs, and lasers. Innovations to present their feasibility, such as ultraviolet detectors, color-discriminating image sensors, neuromorphic devices combining artificial intelligence (AI) applications with optical sensors, and even security applications that are physically unclonable functions (PUFs) or optoelectronic logic circuits have been proposed by exploiting the fundamental advantage of tuning light absorption based on the size of the dots [11,12,13]. As a result, the range of applications using quantum dots is expanding significantly.

In this review, we focus on new device applications that have expanded beyond conventionally reported uses by utilizing the size-dependent bandgap tunability in QDs. In particular, we aim to explore how this characteristic can be used to enhance the functionality of new applications and determine the scope of potential advances in QD-based devices. The paper presents an overview of QDs and their fundamental properties, followed by a comprehensive review of the latest reported technologies in novel device applications, such as optoelectronic circuits, neuromorphic applications, security devices, and UV sensors. Through this analysis, this paper presents a holistic view of the expanding applications of QDs. Furthermore, the challenges and perspectives of QD-based devices and applications are presented, emphasizing how these approaches can be linked to practical and advanced applications.

## 2. Quantum Dots and Their Bandgap Engineering for Optoelectronic Applications

### 2.1. Quantum Dots

QDs are a promising material class for future scientific areas since their discovery by Murray et al. in 1991 [14]. With advances in nanoscience and nanotechnology, nanomaterials are considered essential components in real life. QDs have attracted considerable interest from researchers, owing to their inherent optical and electrical properties [15]. The interesting properties of QDs have made them a theme for the 2023 Novel Prize in Chemistry. QDs, a kind of inorganic semiconducting material with a size scale below 20 nm, have unusual optical and electrical properties attributed to a “quantum confinement effect”, which results in size-dependent bandgap tunability. When NCs are smaller than their Bohr radius (or exciton Bohr radius), their electronic states show discrete energy levels (discontinuous energy states). The exciton Bohr radius can be calculated using the following equation:

(1) $$R_{B}=\varepsilon (\frac{m_{0}}{\mu })a_{0}$$

where *R_B_*, *ε*, and *m*_0_ are the exciton Bohr radius, the dielectric constant of a material, and the mass of a free electron, respectively; *μ* (*m_e_*·*m_h_*/(*m_e_* + *m_h_*)) is the reduced mass of exciton, *m_e_* and *m_h_* are the mass of electrons and holes, respectively; *a*_0_ is the Bohr radius of hydrogen (0.53 Å) [16].

**Figure 1:**
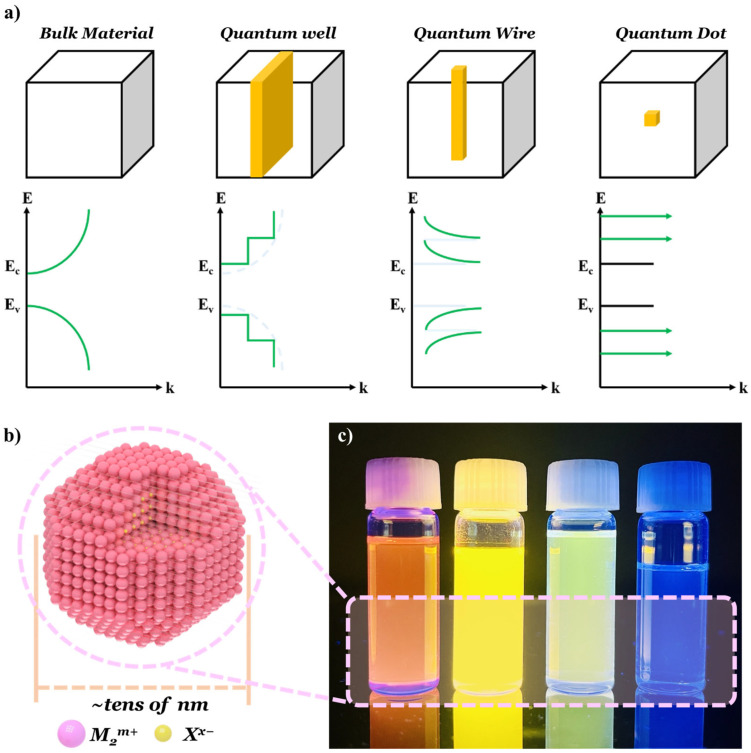
Schematic diagram of (**a**) dimensional-dependent confinement effects and corresponding energy diagrams, and (**b**) quantum dots (QDs) molecular structure. (**c**) Photographs of different energy-emitting QDs (red to blue).

For fundamental insight into the concept, a three-dimensional (3D) nanocrystal (NC) can be imaged as a model system (bulk-state in Figure 1a). A cartesian coordinate system will be used to provide a convenient explanation. When the z-axis of the NC is in the nanoscale (below 20 nm), the corresponding z-axis will also be confined, which results in a stair-like energy state (Figure 1a), and this structure was called a one-dimensional (1D)-confined quantum well [17,18,19]. When the two axes of the NC become smaller, this confinement will lead to two-dimensional (2D)-confined quantum wire structures with further transformed energy states [20,21,22]. If the entire axis of the NC is confined, three-dimensional (3D)-confined QD structures will be formed. In this case, the energy levels of QDs showed fully discrete features, as shown in Figure 1a. These discrete energy levels could be affected by the sizes of NCs, which is called the quantum confinement effect. During these quantum confinement effects, the atomic or molecular orbital could be also confined, which corresponds to the gap between discrete energy states, and the possible orbital (or energy) states can be predicted by numerical methods, such as density functional theory simulation. The aforementioned orbital or energy state can be called “density of state (DOS)”.

The DOS can be defined as the number of orbital states per unit volume with energy between *E* and *dE* as shown in the below equation:

(2) $$g_{i}\left(E\right)dE=\sum_{spin}\sum_{min}\sum_{other}\frac{dk}{{\left(2\pi \right)}^{i}}$$

where, *i* is the number of dimensions, *dk* is the element for differential volume (3D), area (2D), or length (1D) for a surface of constant energy, and the summations are taken over spin, degenerate band minima, and any other mechanisms which are responsible for a degeneracy of electronic states [23].

Moreover, the possibility for the existence of a charge carrier (electron or hole) in each DOS can be predicted by the Fermi–Dirac function. For instance, in the case of superlattice, the distribution of electronic states, or the DOS, is somewhere between that of a quantum well and the bulk semiconductor, and the absorption and emission line shape are greatly affected due to the form of the DOS function [23]. Typically, QDs consist of transition metal ions with their oxidation states (M^m+^) and counter anions with corresponding oxidation states (X^x−^) (Figure 1b). Tamukong et al. investigated the dynamics in the interaction between cadmium selenide (Cdse) QDs and acetate ligands with a variation of excess Cd^2+^ ions, using the DFT calculation [24]. They provided optimal conditions for high optical performance with minimal ligand trap state, which might lead to an optical performance decrease via DOS simulation investigation [24]. Another property of QDs is a fluorescence phenomenon, which can be observed in several semiconductor materials or organic fluorescent dyes. When external energy, e.g., light (photon) or electricity (electron), is introduced into a semiconductor material, an electron positioned in its valence band (VB) is excited to the conduction band (CB) in a femtosecond scale, followed by a rapid (pico- to nanosecond scale) recombination of the excited electron (in the CB) and hole (in the VB) [25], called an exciton (photogenerated electron–hole pair). The charge carrier transition instigated by the incident electromagnetic field can be translated into Rabi oscillation (i.e., Rabi cycle, or Rabi flop), which is affected by lattice inertia [26]. When charges in QDs interact with bulk longitudinal acoustical (LA) phonon, a hybridization between them can occur with dephasing on the picosecond-scale, even under low temperatures, resulting in a probable performance decrease of QD-embedded optoelectronic devices. To understand the dephasing phenomena, studies were conducted on the decoherence of QD spin in a magnetic medium such as diluted magnetic semiconductors (DMSs), in which magnon was (spin wave) incorporated to increase the gyromagnetic factor and accelerate the spin control due to the low gyromagnetic factor of the surroundings. Interestingly, a magnon-incorporated system can offer much favorable control for spin decoherence, which can play a destructive role for QD spins similar to phonon, under low temperature [26]. Nevertheless, the electromagnetic field-induced charge carrier recombination may involve some energy release in the form of a photon; the entire process is called fluorescence, which is a kind of decoherence process. The energy released might correspond to the energy gap between the VB and CB, called the bandgap energy (*E_g_*). QDs can emit various photon energies under external energies due to these quantum confinement effects and fluorescence properties (Figure 1c). Even though there are several reports for the preparation of QDs, the synthetic approaches can be summarized in two different categories, i.e., top–down and bottom–up [27,28]. Table 1 lists the characteristics of each synthetic approach.

The top–down approaches are much simpler processes for preparing QDs. However, some drawbacks exist, such as the lack of product quality, less reproducibility, and inefficient crystalline and morphological structure controllability [28], and the advances in nanoscience have accelerated the intense development of synthetic methods based on bottom–up approaches [28]. With some critical factor modulations of the above synthetic approaches, bandgap-engineered QDs could be realized, and the details will be dealt with in the next section (Section 2.2).

### 2.2. Bandgap Engineering Approaches for Quantum Dot Applications

**Figure 2:**
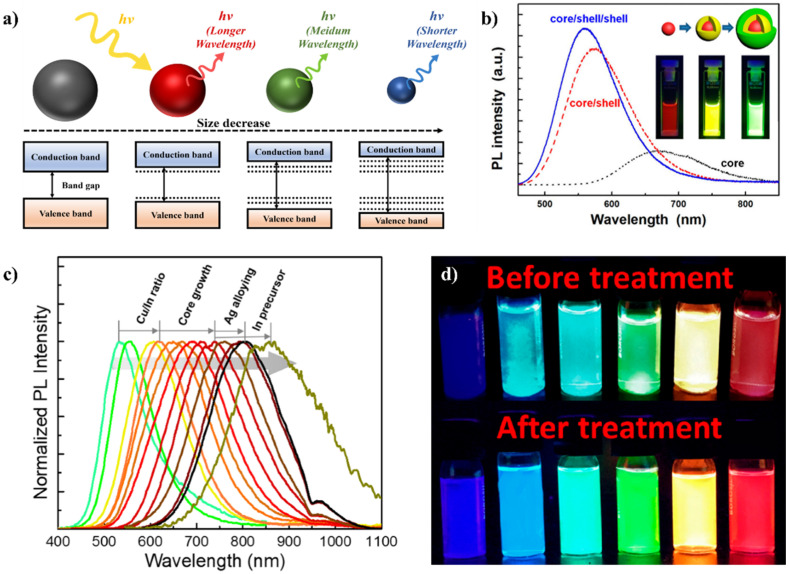
(**a**) Schematic diagram of the size-dependent bandgap tunability of QDs. (**b**) Composite- and structure-dependent bandgap-engineered CuInS_2_ QDs to realize white light-emitting diodes (WLEDs). Adapted with permission from [58] Copyright 2015, American Chemical Society. (**c**) Composition- and doping effect-dependent bandgap engineering to realize broad spectral region CuInS_2_ QDs. Adapted with permission from [59] Copyright 2019, American Chemical Society. (**d**) Realization of full-color capable perovskite QDs (PQDs) with an enhanced physical stability achieved by post-treatment. Adapted with permission from [1] Copyright 2022, American Chemical Society.

In materials science, the energy state of the material is an intrinsic property that cannot be modulated using traditional methodologies. On the other hand, after the discovery of QDs in 1991, the size-dependent bandgap tunability of inorganic semiconductors has been successfully implemented in scientific areas. In particular, QDs are a tremendously attractive material class because of their quantum confinement effect derived from the bandgap engineering phenomena (Figure 2a), which cannot be achieved easily in single-molecule organic fluorescent dyes.

Numerous reports related to various QD compositions were already reported, such as metal-oxide (e.g., ZnO and SnO_2_) [60,61,62,63], II–VI (CdE and ZnE, where E: S, Se, and Te) [14,63,64,65,66,67], IV–VI (PbE, where E: S, Se, and Te) [68,69], I–III–VI (CuInE_2_ and AgInE_2_, where E: S, Se, and Te) [58,59,70,71,72,73], carbon-based QDs (carbon dots (CDs)), graphene QDs (GQDs) [47,74]), and perovskite QDs (PQDs) [1,53,75,76]. Several methodologies have been reported to tune their bandgap energies, e.g., impurity doping [59,65], surface passivation [58], morphological structure modification [75], and size- [59] and composition-dependent modulations [1,59]. This paper does not provide detailed information because the purpose is to introduce QD-embedded optoelectronic device applications. For example, Jang et al. reported white light-emitting diodes (WLEDs) consisting of bandgap-engineered CuInS_2_ (CIS) QD with a broad spectral region (yellow to green), as shown in Figure 2b. They controlled the Cu/In ratio to broaden its bandgap and designed a core/shell/shell structure to induce significant Zn^2+^-ion penetration into the core crystal and complete the confinement by wide bandgap materials (Figure 2b) [58]. Yoon et al. [59] reported a composition- and size-dependent bandgap engineering methodology to produce broad spectral region emissive QDs (Figure 2c). Their approach was focused on three key factors: (i) the composition of transition metal ions (Cu^+^ vs. In^3+^), (ii) the size of the QDs, and (iii) impurity doping into QDs (Ag^+^), and a successful preparation of visible-to-near infrared (NIR) window emissive QDs was achieved [59] (Figure 2c). In 2022, Mishra et al. reported various color-emissive PQDs (Figure 2d). Moreover, the prepared PQDs were post-treated by ascorbic acid for the enhancement of optoelectronic performance. The PQDs showed halide anion-dependent emissive properties, and they successfully allowed the bandgap modulation of PQDs from violet (410 nm, ~3.0 eV) to red (680 nm, ~1.82 eV) [1] (Figure 2d). Such a bandgap engineering feature is a powerful tool for the variation of material properties, which can lead to various applications of QDs in biological imaging [77], biomedical therapeutic applications [78,79], photopolymerizations [80,81], photoelectrochemical hydrogen production [82,83] and carbon dioxide reduction [84,85], sensors [86,87], and optoelectronic devices [6,7,8,9]. This review focuses on the QD-embedded optoelectronic device applications with bandgap engineering. The themes of device applications are divided into three categories: (i) light-detection devices (or photodetectors, PDs), (ii) optoelectronic semiconductor devices, and (iii) QD-assisted security devices (e.g., physically unclonable function (PUF) devices). Section 3 provides a more detailed explanation.

## 3. Device Applications of Bandgap Engineered Quantum Dots

### 3.1. Deep-Ultraviolet (DUV) Photodetectors (PDs)

Photodetectors (PDs) are a kind of sensors that detect an incident light signal (input) and transduce it into an electrical signal (output) for the distinction and quantization of the light (Figure 2a) [88].

**Figure 3:**
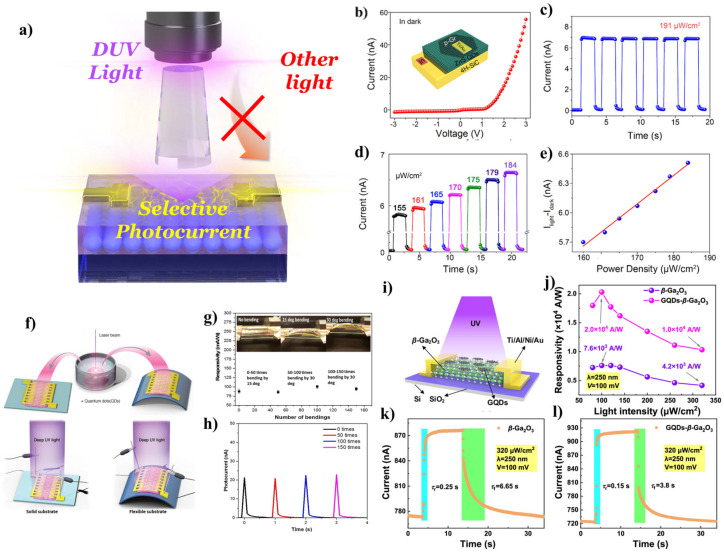
(**a**) Schematic diagram of DUV PDs based on ultra-wide bandgap QDs with specific sensitivity in DUV wavelengths. (**b**) Current–voltage and (**c**) current–time characteristics of ZnS QD-based DUV PDs. (**d**) Light intensity-dependent photoresponse of the device under 250 nm DUV irradiation and self-powered operation condition. (**e**) Extracted photocurrent variation according to the light power density. Adapted with permission from [89] Copyright 2019, American Chemical Society. (**f**) Schematic diagram of the synthetic and measurement process of ZnO QD-based DUV PDs. (**g**) Measurement of the responsivity of the flexible device as a function of the number of bending cycles. The inset images illustrate the degree of bending of the device. (**h**) Photocurrent generation after different numbers of bending cycles at a specific angle. Adapted with permission from [95], Copyright 2018, Elsevier. (**i**) Schematic diagram of DUV PDs based on *β*-Ga_2_O_3_ and GQDs. (**j**) Spectral responsivity of bare *β*-Ga_2_O_3_ and GQDs/*β*-Ga_2_O_3_ PDs according to the light intensity. Transient photoresponse of (**k**) *β*-Ga_2_O_3_ and (**l**) GQDs/*β*-Ga_2_O_3_ PDs under DUV irradiation. Adapted with permission from [96] Copyright 2022, American Chemical Society.

Among many spectral range recognition systems, deep-ultraviolet (DUV) PDs have been emerging research topics because of their complex fabrication and inefficient performance [89]. For DUV PDs, ultra-wide bandgap semiconductor materials should be implemented. However, wide bandgap materials are relatively rare [90], which could hinder PD design and development [89]. Several efforts have been made to prepare active layers capable of high-energy endurance and transduce them into electricity. Zhang et al. reported a DUV PD system based on a carbon-doped hexagonal-boron nitride (h-BCN) atomic layer as a DUV active layer to enhance performance and responsivity [91]. By the realization of the atomic layer structure of h-BCN, they enhanced the performance compared to the counterpart of the h-BCN nanosheet. Tanaka et al. [92] reported DUV PDs based on MgGa_2_O_4_ films deposited using a relatively low-temperature pulsed laser deposition (PLD) technique. They optimized MgGa_2_O_4_ film preparation using a substrate temperature-dependent PLD process. Furthermore, Lee et al. [93] reported a flexible DUV PD by implementing *β*-Ga_2_O_3_ amorphous film onto a polyimide substrate. They used an atomic layer deposition (ALD) technique to fabricate flexible DUV PD with a high rejection ratio of ~104 (R_220nm_/R_350nm_). Nonetheless, the DUV PDs usually consisted of metal oxide-based systems and were fabricated using deposition methods. In this regard, QDs could be a simple solution for these issues because of their bandgap tunability and solution processability (Figure 3a). For example, zinc sulfide (ZnS), a typical n-type semiconductor material with a bulk bandgap energy of ~3.76 eV [94], could be implemented in DUV PD applications using a quantum confinement effect [86]. The bulk bandgap energy of ZnS cannot cover the DUV spectral region (DUV: 4.42~6.2 eV), but bandgap widening occurred when the ZnS size was on the nanoscale (below 10 nm), which resulted in the coverage of the DUV region. Huang et al. reported ultrafast photovoltaic type DUV PDs using hybrid zero-/two-dimensional heterojunctions consisting of a p-type graphene/ZnS QDs/4H-SiC (Figure 3b–e) [89]. The device exhibited excellent selectivity for the DUV spectral region and showed an ultrafast response rate (rise time, 28 μs; decay time, 0.75 ms). In this report, the authors successfully used the quantized ZnS QDs in PIN junction-based architectures to realize PDs (Figure 3b–e).

Zinc oxide (ZnO), another n-type semiconductor material with a bulk bandgap of ~3.37 eV [97], could also be implemented into DUV PD applications by the quantum confinement effect [95]. Roqan et al. group reported high-performance solar-blind flexible DUV PDs using ZnO QDs as active materials for DUV light (Figure 3f–h). They synthesized ZnO QDs using the femtosecond-laser ablation in liquid (FLAL) technique. They also utilized carbon-doping into ZnO QDs to enhance the stability and conductivity. The fabricated DUV PDs based on C-ZnO QDs onto flexible substrates showed high responsivity, fast response time, and stable switching performance. In addition, the prepared QDs were unaffected by water or oxygen, suggesting an enhanced stability of the devices [95]. Even the metal-based composites are efficient probes for detecting DUV light. Carbon-based materials also could be used in DUV PD systems [96]. Carbon-based materials have attracted considerable attention from researchers because of their distinct properties on the nanoscale. One-dimensional confined graphene (2D) structures possess exceptional conductivity. When graphene (or carbon) was confined in all 3D axes, it exhibited a similar quantum confinement effect with transition metal-based QDs. These materials were called carbon dots (CDs) or graphene QDs (GQDs). The C-QDs could be implemented in DUV PD systems because of the quantum confinement effect and other factors. Lu et al. reported a hybrid system consisting of GQDs (0D) and *β*-Ga_2_O_3_ (quasi-2D) for high-performance DUV PDs [96]. The pristine *β*-Ga_2_O_3_ were considered solar-blind (200–280 nm) PDs (Figure 3i–l), but metal–semiconductor–metal (MSM) PDs fabricated with *β*-Ga_2_O_3_ suffered from a low photoresponsivity, slow response rate, and a narrow detection wavelength [98]. The authors introduced GQDs to solve these issues via quantum confinement effects. The quantized GQDs showed a high extinction coefficient and high surface area. In addition, GQDs can exhibit synergetic effects by incorporating 2D materials (*β*-Ga_2_O_3_) [99]. The authors implemented these properties into DUV PD applications with ultrahigh responsivity (R of ~2.4 × 10^5^ A/W), a large detectivity (D* of ~4.3 × 10^13^ Jones), an excellent external quantum efficiency (EQE of ~1.2 × 10^8^%), and a rapid photoresponse (150 ms).

### 3.2. Dual Band (DB) Photodetectors (PDs)

**Figure 4:**
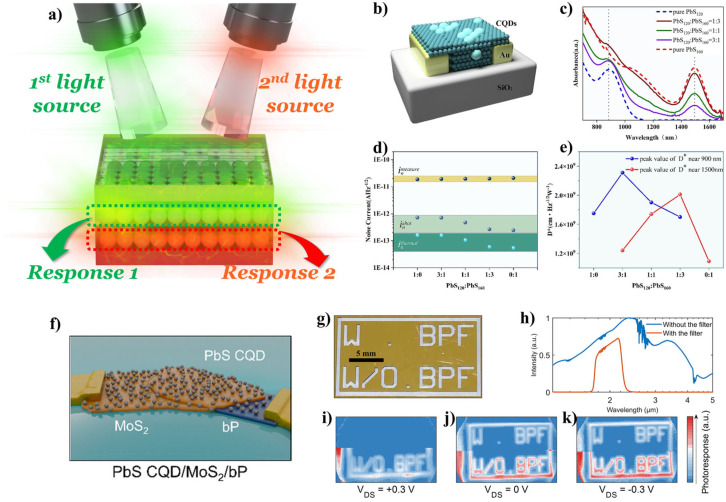
(**a**) Schematic diagram of dual-band (DB) PDs. (**b**) Device configuration of a PbS QD-based photoconductor-type PD. (**c**) Absorption profiles of mixed PbS QDs varied with different weight ratios. (**d**) Average noise current and (**e**) specific detectivity values of PbS QD-based PDs with different weight ratios. Adapted with permission from [102] Copyright 2024, Elsevier. (**f**) Conceptual schematic diagram of the bias selective DB PD based on PbS QDs/MoS_2_/black phosphorous (bP) stacked layers. (**g**) Photograph of the mask pattern fabricated on a glass slide with Au. (**h**) Normalized spectral density of the broad-spectrum IR source according to the presence of the band-pass filter. The band-pass filter restricts the illumination with a spectral region of λ = 1.7–2.4 μm. IR images extracted from the PbS CQD/MoS_2_/bP PD, shown (top) with a band-pass filter and (bottom) without it, under (**i**) forward bias with drain-source voltage (V_DS_) = 0.3 V, (**j**) zero bias with V_DS_ = 0 V, and (**k**) reverse bias with V_DS_ = −0.3 V. Full images are only obtainable with the band-pass filter under zero and reverse bias conditions. Adapted with permission from [100] Copyright 2023, American Chemical Society.

Infrared (IR) region photodetection applications are also considered essential devices in the fast-expanding application space, spanning communication, computing, imaging, and sensing [100]. Polarization-dependent intensity information, or intensity discrimination in multiple IR spectra bands, were realized using single IR PDs [100]. Between them, by the utilization of the latter property, some newly emerging applications, e.g., multiband imaging, remote sensing, temperature/flame detection, spectral reconstruction, and object identification, were proposed [100]. The simplest form, known as “two-color” or “dual-band (DB)” PDs, was typically achieved by stacking two IR photodiodes in a tandem or back-to-back structure [101]. To achieve IR spectral response, ultranarrow bandgap semiconductor materials are essential, but these materials are limited to several compounds, such as mercury–cadmium telluride (MCT, or HgCdTe), and indium arsenide (InAs) alloyed with gallium (Ga) or antimony (Sb). Continuous effort has been made to develop device architectures and materials that could overcome these limits because there are some obstacles in using DB PDs in the industrial fields, e.g., high fabrication cost and complexity, the need to consider lattice matching, surface passivation, and high noise at room temperature [102]. In this aspect, QDs could be the simplest and most effective solution because of their size- or composition-dependent bandgap tunability and simple preparation methods. For example, DB PDs could be realized by mixing two types of QDs with different size- or composite-induced bandgap-engineered QDs (Figure 4a). Wen et al. reported a mixed QD film for DB IR PD applications [102]. They prepared a mixed-lead sulfide (PbS) QDs film with different sizes (i.e., different bandgap energy) using a spin coating technique and implemented the film into DB PDs (Figure 4b) [102]. By optimizing the QD ratio, they obtained a DB PD with a peak response at 900 nm (responsivity: 0.18 A/W, and specific detectivity: 2.38 × 10^9^ cm∙Hz^1/2^/W), for the NIR spectral region, and 1500 nm (responsivity: 0.17 A/W, and specific detectivity: 2.11 × 10^9^ cm∙Hz^1/2^/W), for the shortwave IR (SWIR) spectral region, respectively (Figure 4c–e). Bullock et al. reported bias-selectable DB IR PDs using PbS colloidal QDs and black phosphorous (bP) [100]. They used mixed-dimensional device switches between two detection mechanisms consisting of a phototransistor (PbS QDs/MoS_2_) for the spectral response peak in the SWIR centered at 1.45 μm and a photodiode (bP/MoS_2_) for the spectral response peak in mid-wave IR (MWIR) under zero-bias, respectively (Figure 4f–k) [100]. Moreover, the system was further integrated into complementary metal-oxide-semiconductor (CMOS) read-out chips, suggesting a potential technological pathway forward. Tang et al. reported an impressive research result [103]. The authors realized a DB IR imaging system using stacked colloidal QD photodiodes in the spectral region of SWIR and MWIR by introducing two different sizes of mercury telluride (HgTe) QDs [103]. A two-stacked photodiodes system was used in a back-to-back configuration. They showed SWIR (spectral) and MWIR (thermal) imaging and bias-switchable SWIR/MWIR sensing performance with a rapid switching rate at modulation frequencies up to 100 kHz, with a D* value over 10^10^ jones at cryogenic temperature [103].

### 3.3. Bandgap Engineered QDs for Emerging Optoelectronic Computing Devices

**Figure 5:**
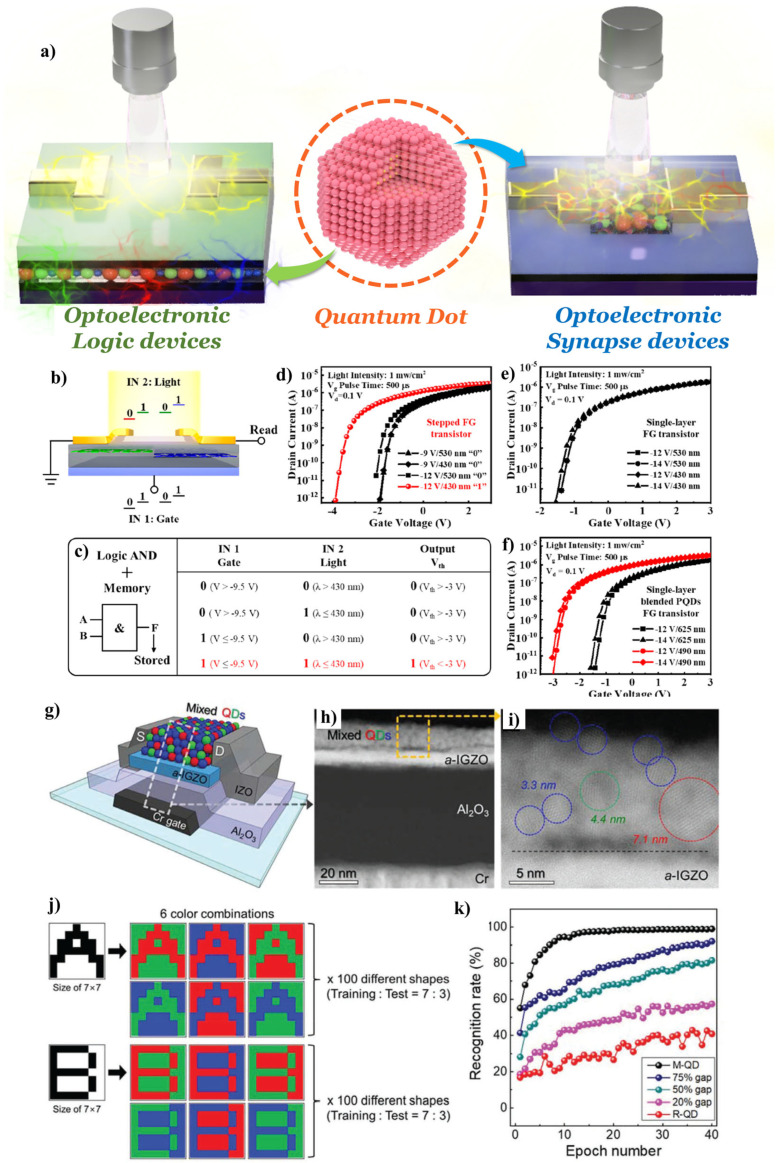
(**a**) Schematic diagram of bandgap-engineered QD-assisted optoelectronic devices for logic (**left**) and synapse (**right**) applications. (**b**) Schematic diagram of the device architecture, optoelectronic logic gate operation, and (**c**) truth table. (**d**–**f**) Transfer characteristics of devices varied with device architectures: (**d**) stepped floating gate (FG), (**e**) single-layer FG, and (**f**) single-layer blended PQDs FG. Adapted with permission from [104] Copyright 2022, American Chemical Society. (**g**) Schematic device structure of the M-QD/a-IGZO phototransistor-based artificial synapse device. (**h**) Cross-sectional STEM image of the M-QD/a-IGZO phototransistor, and (**i**) a magnified view of the M-QD layer within the phototransistor. (**j**) Examples of RGB color combinations from the dataset. (**k**) Recognition rate of color pattern images during the training epochs. Adapted with permission [105] Copyright 2022, Wiley-VCH GmbH.

From the viewpoint of optoelectronic devices, the main advantage of QDs is the controllable absorption range for the target wavelengths through size modulation. Emerging optoelectronic computing or logic devices, such as logic gates and neuromorphic devices, using light as input signals or external stimuli, have been widely reported [104,105]. The bandgap-engineered QDs combined with optoelectronic devices can provide specific light–matter interactions, resulting in wavelength-dependent outputs, as shown in Figure 5a. Ding et al. reported a bi-functional optoelectronic logic and memory device based on the floating gate architecture [104] (Figure 5b–f).

As represented in Figure 5b, cesium lead halide (CsPbX_3_, X: Cl^−^, Br^−^, and I^−^) perovskite quantum dots (PQDs) with different energy bandgaps were introduced to an amorphous indium gallium zinc oxide (a-IGZO) transistor as a light-sensitive floating gate (Figure 5b). The narrow and wide bandgap PQDs were fabricated with the unique stepped floating gates structure through a vapor deposition method (Figure 5b). Depending on the wavelength of the optical input signal, the light-induced charge trapping effects varied according to the bandgap of the PQD floating gates. The trapped charges lead to threshold voltage shifts, resulting in optoelectronic memory operation. Furthermore, the proposed device exhibited optoelectronic ‘AND’ logic gate behavior using electrical and optical inputs (gate and light) in a single transistor scheme (Figure 5c–f). Park et al. proposed optoelectronic synaptic transistor arrays based on light-sensitive QDs in 2022 [105] (Figure 5g–k). They deposited a mixed QD layer directly on the IGZO transistor (Figure 5h–j). The mixed QD layer comprised different bandgap QDs for a multispectral photodetection. Moreover, they mimicked a biological photoreceptor system using the mixed QDs photoabsorbers with precise RGB ratio control. They enabled full-range visible color recognition with high photo-to-electric conversion efficiency. In addition, multiple nonvolatile-to-volatile memory conversion was implemented into the system via adjustable synaptic plasticity, modulated by gate bias, resulting in chromatic control in the artificial photonic synapse. A 7 × 7 pixelated photonic synapse array with outstanding color image recognition was successfully realized based on adjustable wavelength-dependent volatility conversion (Figure 5g,k) [105].

### 3.4. Physically Unclonable Functions (PUFs)

With a rapid expansion of cutting-edge innovations for human life convenience, the widespread use of counterfeit products raises significant economic, social, and health concerns [106,107]. Traditional anti-counterfeiting methods are generally accompanied by attaching security labels to products. Various types of labels are used for anti-counterfeiting purposes, such as watermarks, holograms, graphical barcodes, and security inks, and the fundamental principle of these anti-counterfeiting methods is based on the production of a distinct response (output) from the security label when exposed to a specific challenge (input). For example, a graphical logo that appears under ultraviolet (UV) light is a typical example used in banknotes. Recently, the ease of accessing information has enabled counterfeiters to replicate security labels [106]. In this regard, physically unclonable functions (PUFs) have emerged as a highly promising solution to this issue and have attracted considerable attention from researchers. Recent studies focused on using the randomness generated during fabrication and the unique properties of materials. The optical properties of materials may be crucial for developing efficient PUF systems.

**Figure 6:**
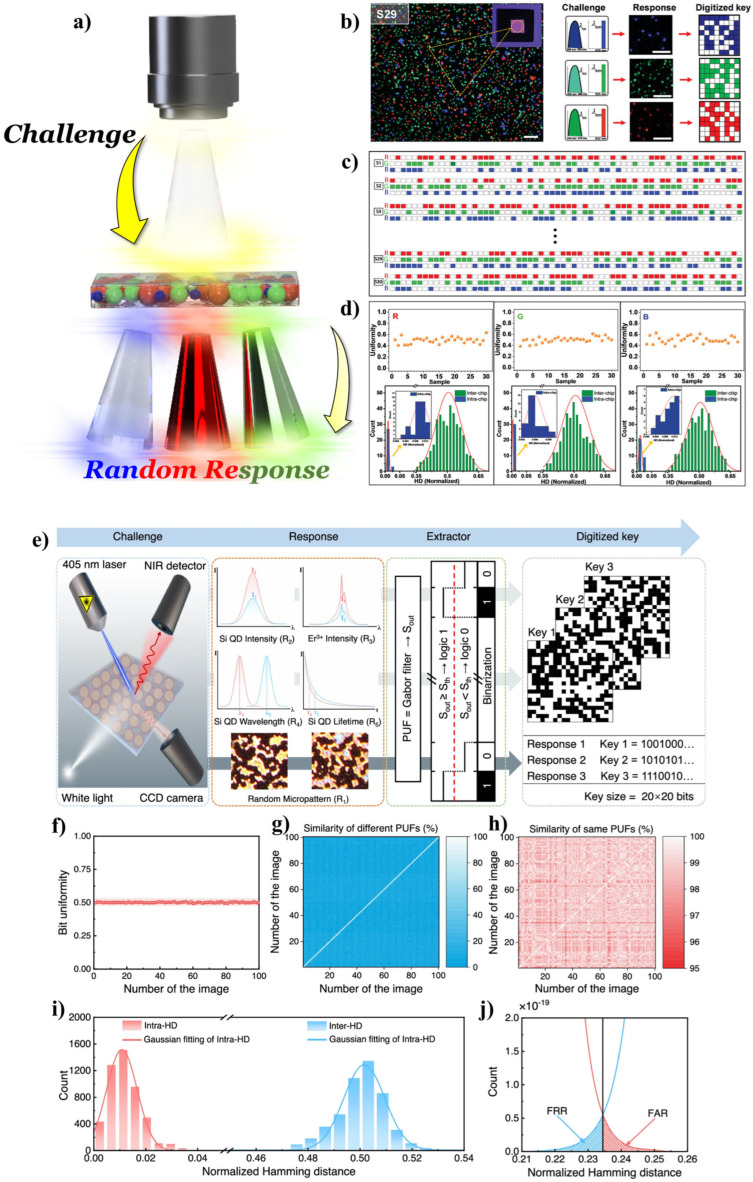
(**a**) Schematic diagram of bandgap-engineered QD-assisted security devices (PUFs). (**b**) Schematic diagram of the random pattern extraction process with the QD-based optical PUF devices. (**c**) Demonstration of three distinct challenge-response pairs, each optimized for the individual components of a single PUF, along with the unique PUF keys produced from these pairs. Scale bars represent 100 μm. (**d**) Bit uniformity, intra-Hamming distance (HD), and inter-HD analysis results of the random codes generated by tattoo-like multicolor PUFs. Adapted with permission from [106] Copyright 2023, Wiley-VCH GmbH. (**e**) Challenge-response authentication process of the optical PUF device. (**f**) Bit uniformity of binary bits extracted from 100 images. (**g**) Pairwise comparison of 100 different PUFs by the per-pixel binary encoding of images captured from various positions. (**h**) Pairwise comparison of 100 identical functions using the per-pixel binary encoding of images captured from the same position. (**i**) Distribution of normalized HD for inter-PUF (Inter-HD) and intra-PUF (Intra-HD) comparisons. (**j**) Magnified views of Inter-HD and Intra-HD. Adapted with permission from [107] Copyright 2024, Springer Nature.

Optical PUFs have rapid and simple read-out properties, making them highly desirable devices. In particular, fluorescent materials have attracted considerable attention because of their unique absorption and emission wavelengths, together with other characteristics such as fluorescence lifetime and PL quantum yield (PLQY), which offer various options in the design and manufacture of unclonable function keys. Regarding these features, QDs could be candidates for anti-counterfeiting materials because of their specific challenge-response mechanisms, which could be modulated by the quantum confinement effect. In addition, the solution processability of QDs makes them fascinating materials for PUF device applications because fluorescent PUFs have been developed by an inkjet printing technique [108]. For example, bandgap-dependent challenges could be generated using bandgap-engineered QDs, which are invisible in the ambient environment. Moreover, diverse security devices can be generated, resulting in much harder-to-counterfeit patterns (Figure 6a).

Liang et al. fabricated PUF keys using a femtosecond laser ablation (FsLA) technique to produce random patterns in micro- and nanoscales [109]. They produced a three-color QD film onto the glass substrate via a spin-coating process that was patterned using the FsLA technique [109]. Unpredictable random behavior occurred (e.g., curvature of the edges) when the film was irradiated with a femtosecond laser to remove the QDs, and they employed the randomness in PUF keys. Moreover, deep learning algorithms were used to evaluate the authentication of PUF keys. The results showed good performance with simple, rapid, and efficient features [109]. Kiremitler et al. reported tattoo-like multicolor PUFs using an electro-spraying technique (Figure 6b–d) [106]. They used the inherent features of the electro-spraying technique, i.e., electrohydrodynamic instabilities, to render the randomness for unclonable functions. In addition, RGB-emissive QDs were implemented into their system to achieve the high-encoding capacity for PUFs. The additive nature of electro-spraying allowed a direct deposition of these QDs on the same substrates, facilitating multicolor PUFs. They decoupled the fabrication process from the applied objects by combining them with tattoo approaches, greatly relaxing the constraints in application. They facilitated the advantages of QDs, e.g., challenge-dependent response and high photophysical stability, and tattoo approach (low cost, mass-producible, and non-destructiveness), for the realization of PUF keys with a high-level of stability and security (Figure 6b–d) [106]. Liu et al. reported inkjet-printed unclonable QDs fluorescent anti-counterfeiting labels with artificial intelligence authentication [108]. They fabricated security ink using three types of QDs (R-, G-, and B-QDs) and patterned the security product labels by inkjet printing. At the patterning steps, poly(methyl methacrylate) (PMMA)-modified hydrophilic surfaces offered critical factors to prevent the coffee-ring effect at a solvent evaporation process by providing stochastic pinning points. These stochastic surfaces eventually render randomness for unclonable functions. In addition, artificial intelligence (AI, specifically deep learning) was used for the security label authentication concept and exhibited an accurate and robust decoding of unpredictable and unrepeatable patterns. Using the three different QDs, they satisfied the low-cost, mass-producible, nondestructive, diverse full-color pattern design capability that was unclonable and convenient for security device fabrication and authentication [108]. Beyond optical PUF devices, Wang et al. reported CMOS technology-integrated optical PUFs [107]. They synthesized erbium (Er)-doped silicon (Si) QDs using a nonthermal plasma synthesis technique and fabricated PUF keys onto traditional Si wafers post-treated to produce inverted pyramid arrays (Figure 6e). Lanthanide-metal ion doping was also used for bandgap engineering. It is well-known that lanthanide-metal ion doping into template nanoparticles (NPs) can cause a photon up-conversion because of their relatively less confined *f*-orbitals. The authors successfully utilized this property in the PUF system (Figure 6e). Such all-Si-based PUFs could realize five in situ optical responses encompassing micropattern imaging, the photoluminescence (PL) intensity of Si QDs and Er^3+^, PL wavelength, and the PL lifetime of Si QDs in a single pixel, which can instigate ultrahigh information entropy (up to 2.32 bits/pixel). In their study, a finite-difference time-domain (FDTD) simulation showed that the Si meta-surface and Er-Si QDs are robustly coupled because of the radiation field and Purcell effect, resulting in a position-dependent optical response (Figure 6f–j) [107].

## 4. Obstacles and Overcoming Strategies

This study introduced various research reports related to the device applications of QDs. Nevertheless, several obstacles must be solved before practical applications to realize high-performance QD-embedded devices and further applications, such as biological sensing and imaging, photovoltaics, biomedical photodynamic therapies, and photocatalysts. These limitations were subdivided into three categories: (i) patterning technologies for QD layers, (ii) the stability of materials, and (iii) the toxicity of heavy-metal ions:

(i) The solution processibility of QDs offers the facile modality of device fabrication processes. Nevertheless, to operate QD-embedded devices, the QD layers need to be integrated into the electronic circuits. Despite the several reports of QD integration into electronic circuits, the patterning technology of QD layers for electronic circuits is still an un-pioneered area [110,111,112,113,114,115,116]. Fortunately, 3D inkjet printing and spray coating techniques could realize solution phase patterning because of the advances in additive industries. However, the lack of pattern resolution is a remaining issue in these fields [110,111,112]. Some approaches for patterning QD layers have been reported, e.g., solid-phase transfer printing [110,111,112] and light-driven ligand cross-linking techniques [116]. Solid-phase transfer printing is a much more adaptable approach because of its less affection toward QD film performance [113,114,115], but specific conditions, such as a PDMS-based sacrificial substrate and the hydrophobic surface properties of QDs, must be addressed. The light-driven ligand cross-linking method is based on the reactive ligand cross-linking process under light irradiation [116], and this technique can open a new pathway for patterning QDs due to the high-resolution patterns compared to conventional additive 3D printing techniques [116]. However, a complex surface modification for cross-linking behavior and its ambient stability must be resolved.

(ii) QD film stability is another obstacle to realizing QD-embedded electronic circuit devices and other scientific and industrial fields. Most QDs have many defects on their surfaces, leading to the degradation of the QD film. QD synthesis involves several surface passivation methodologies with an inorganic layer [117,118], halide anions [119], or relatively long (carbon number > 10) aliphatic hydrocarbon ligands to suppress the Ostwald-ripening effect, which affects the quantum confinement effect [120]. Among them, the most predominantly employed approach is long aliphatic hydrocarbon ligand passivation due to its easy processibility; however, these ligands can result in low performance in charge carrier transport, and to solve this problem, tailoring surface properties could be considered a promising approach to enhance device performance [121,122]. Among various tailoring methods, a direct surface ligand exchange process with shorter aliphatic or aromatic ligands could enhance charge transport efficiency. However, this process has a side effect of appealing surface defects onto the QD surfaces, leading to QD film degradation. In addition, QDs have a high surface-to-volume ratio that can have a synergetic effect with these surface defects, accelerating QD film degradation. To solve this issue, newly emerging strategies for efficient ligand cleavage without forming surface defects should be developed.

(iii) Heavy-metal ion toxicity might be considered a fundamental and inherent issue related to QD application in numerous fields [11,113]. Cadmium (Cd), lead (Pb), and mercury (Hg) are the typical QD components. These components are well-known toxic elements. For example, according to the Agency for Toxic Substances and Disease Registry, (ATSDR) in 2022, these elements were listed in the top 10 (Cd: 7th, Pb: 2nd, and Hg: 3rd), and most investigated QDs contain these heavy-metal ion-based chalcogenides (CdE, PbE, and HgE, where E: S, Se, and Te) [3,8,90,91,92,93,94,95,96,97,98,99,100,101,102,103,123]. In particular, until now, Pb and Hg are dominant components beyond the NIR spectral region (over ~800 nm) because of their efficient coverage for that region [100,101,102,103,123]. These elements should be substituted with other less toxic components to realize industrial applications. Several efforts have been made to achieve heavy-metal-free QDs for NIR spectral region, e.g., CDs, GQDs, CuInS_2_, and CuInSe_2_ QDs [11,124]. Nevertheless, the lack of an IR region is still a remaining challenge.

## 5. Conclusions

This review introduced several emerging optoelectronic device applications that involved QDs in their systems. Owing to the quantum confinement effect-derived bandgap tunability, QDs can be implemented into several device applications such as photodetection systems, optoelectronic logic and synaptic devices, and security devices. Recently, extreme spectral ranges, such as the DUV, NIR, and IR regions, could be detected by QD-embedded PD systems. In addition, their homogeneous junction-like systems with different bandgap energies were used in floating gate logic devices and synapse-mimicking artificial systems. Furthermore, the different bandgap energy QDs have a high potential for anti-counterfeiting applications. Further improvements in pattering technologies, the enhancement of material stabilities, and the substitution of highly toxic heavy-metal components are expected to realize real applications.

## Figures and Tables

**Table 1:** A comparison between top–down and bottom–up approaches for the preparation of QDs.

Approaches	Top–Down	Bottom–Up
Features	From bulk to nanoscale, physical atomization mechanisms	From atomic to nanoscale, chemical growth mechanisms
Advantages	Relatively fewer complex systems [29]; bulk scale producibility [30]	High-quality products, high reproducibility, and various composition producibility
Disadvantages	Poor optical and electrical performances, poor reproducibility, and less effective geometric controllability [28]	Complex synthetic design; limitation for mass-production
Methods	Sonication exfoliation [29,31,32,33,34], heat treatment [35,36], electrochemical exfoliation [37,38,39], mechanical exfoliation [40,41,42], and laser ablation [43,44,45]	Solvothermal (or hydrothermal) [46,47,48], microwave-assisted solvothermal [49,50,51], hot-injection [52,53], and heating-up [54,55,56,57]

## Data Availability

Not applicable.
